# Comparison of the effect of hemihydrate calcium sulfate granules and Cerabone on dental socket preservation: An animal experiment

**DOI:** 10.15171/joddd.2018.037

**Published:** 2018-12-19

**Authors:** Naser Sargolzaie, Mehrnaz Rafiee, Hamideh Salari Sedigh, Reza Zare Mahmoudabadi, Hooman Keshavarz

**Affiliations:** ^1^Dental Materials Research Center, Mashhad University of Medical Sciences, Mashhad, Iran; ^2^Periodontist, Mashhad, Iran; ^3^Department of Clinical Sciences, Faculty of Veterinary Medicine, Ferdowsi University of Mashhad, Mashhad, Iran3Department of Clinical Sciences, Faculty of Veterinary Medicine, Ferdowsi University of Mashhad, Mashhad, Iran; ^4^Department of Oral and Maxillofacial Pathology, School of Dentistry, Mashhad University of Medical Sciences, Mashhad, Iran; ^5^Department of Community Oral Health, School of Dentistry, Mashhad University of Medical Sciences, Mashhad, Iran; ^6^Dental Research Center, Mashhad University of Medical Sciences, Mashhad, Iran

**Keywords:** Animal experiment, calcium sulfate, Cerabone, dog, socket preservation

## Abstract

***Background.*** Early bone loss due to tooth extraction can be significantly reduced by socket preservation. The aim of this study was to compare the in vivo effects of hemihydrate calcium sulfate granules (an alloplastic material) and Cerabone (a bovine-derived xenograft) on socket preservation in dogs.

***Methods.*** Six male Mongrel dogs were randomly divided into 2 groups (n=3) for sacrificing and histological evaluation 4 and 8 weeks after a surgery. The second and third premolars on both sides of the lower jaw were extracted surgically. The sockets on one side were filled with Cerabone, and with calcium sulfate on the opposite side. In the slides, the ratio of the area of newly formed bone to the area of the entire cavity, and the ratio of the area of fibrous connective tissue to the area of the entire cavity were measured. The presence of inflammation was also examined. Wilcoxon signed-rank test, Sign test and McNemar test were used for statistical analyses (ɑ=0.05).

***Results.*** The means of new bone proportion were 11% and 8% for Cerabone and calcium sulfate, respectively (P=0.58). The means of connective tissue proportion were 29% and 33% for Cerabone and calcium sulfate, respectively (P=0.72). No inflammatory cells were observed in the Cerabone group, although 50% of the samples in the calcium sulfate group showed inflammation (P=0.50).

***Conclusion.*** The effects of calcium sulfate and Cerabone on socket preservation in dogs on bone formation, fibrous connective tissue and inflammation levels were not significantly different at 4- and 8-week postoperative intervals.

## Introduction

The form of the alveolar bone is dependent on teeth, and the evolution of the alveolar process is determined by the form and axis of tooth eruption.^1^ Thus, it is not surprising that the alveolar process undergoes different amounts of bone resorption following tooth loss.^2^ It has been shown that within 12 months after tooth extraction, the ridge width is reduced by an average of 50%, and two-thirds of this change occur in the first three months. On average, this reduction is about 5−7 mm and the same in all sites of the mouth.^3^

Bone density and quantity are the primary conditions for dental implantation, and significant loss of bone tissue leads to escalated complexity of implantation in a prosthetically appropriate site.^2^ Preservation of the alveolar process after tooth extraction can be predictable. This allows the implantation to be in a satisfying position in terms of beauty and function.^4^ Socket preservation dramatically diminishes the loss of width and height of the ridge after tooth extraction.^2^ Thus, it can diminish the need for extensive surgical interventions and increased treatment costs in the future.^5^

For socket preservation, different methods have been recommended involving the use of different bone grafts.^6^ Types of bone grafts can be divided into four general groups of autogenous grafts, allogeneic grafts, xenogeneic grafts and alloplastic materials.^7,8^ Allografts and xenografts are associated with the potential for transmission of pathogens.^9,10^ To avoid biological risks, these substances undergo steps that have negative effects on their osteogenic and osteoinductive properties. These steps can also reduce their structural cohesion and lead to graft failures.^9,11,12^ In fact, alloplastic materials have been produced to minimize such problems.^13^

Calcium sulfate as an alloplastic material is biologically neutral and a highly biocompatible material. One of the easiest methods of manufacture, and the longest clinical history (more than 100 years) belong to this material.^14^ The primary benefits of calcium sulfate include easy handling, adsorption by osteoclasts, and the binding and deposition of osteoids by osteoblasts.^15^ On the other hand, an important feature which bone grafts should have in order to be able to properly repair bone is the appropriate degradation rate. This rate should be similar to that of bone formation. The mass of the bone graft should allow the formation of bone within itself, and this process will continue to complete replacement of bone.^16^ As the degree of hardness increases, the rate of degradation and resorption diminishes.^17^ Reports differ in relation to the calcium sulfate degradation period, depending on the size of the site, blood circulation of the area, and the tested model.^18^

With the aim of slowing down the resorption rate of calcium sulfate, Sargolzaie et al^19^ prepared hemihydrate calcium sulfate granules with a size of 500−700 µm and suitable hardness of dehydrate calcium sulfate powder, which was used to repair bone defects in the skull of rabbits. The results of that study showed that hemihydrate calcium sulfate granules had good biocompatibility and promoted bone healing.

The aim of this study was to compare the in vivo effects of hemihydrate calcium sulfate granules and Cerabone on socket preservation in dogs.

## Methods

Six adults, mixed-breed (Mongrel) male dogs weighing 15−25 kg with the mean age of 2 years were housed in individual cages under the supervision of a veterinarian at Animal Research Center of Mashhad Dental School for two weeks before any surgery. The environment was precisely controlled for light (12 hours light: 12 hours dark photoperiod) and temperature (20±2°C) with free access to water and commercially balanced dry food (made in France). Anti-parasitic drugs and vaccination were administered during their housing. All the steps for each animal were specifically performed and recorded.

The animals were numbered from 1 to 6. Three of them (randomly selected using a computer-based method) were sacrificed four weeks after the surgery. Three other dogs were sacrificed eight weeks after the surgery. In each of the above-mentioned groups (each group with three dogs), the surgery was performed in order of the assigned numbers.

### The animal model of surgery

The lower jaw of dogs on each side has four premolar teeth. The second and third premolars have two divergent roots, and the extraction of each of them results in two identical cavities in size and position on the jaw sides. Since the second and third premolars have approximately the same dimension, the cavities obtained by extracting them are similar.

General anesthesia was induced after 12 hours of fasting state. Conventional infiltration anesthesia (2% lidocaine, Epinephrine: 0.00001, produced by Darou Pakhsh Co., Iran) was also administered in the mental foramen. After that, the oral cavity of the animal was washed with 0.12% chlorhexidine mouthwash, and then on both sides of the lower jaw, the second and third premolar teeth were extracted. To prevent the remaining bony walls being damaged, the roots were separated by drilling and extracted independently by forceps. For better visibility of the bony walls and also the possibility of the tension-free covering of cavities after insertion of materials, the buccal and lingual flaps were elevated beyond the mucogingival junction.

In each group of dogs, in the first surgery (surgery on the dog with the lowest number) calcium sulfate (granular form, 500−700 µm in diameter) was placed in the cavities of the right side of the mandible, and Cerabone (a bone graft with bovine origin, 0.5−1 mm in diameter, made in Germany) in the cavities of the left side of the mandible. In the second surgery, Cerabone was placed in the cavities of the right side, and calcium sulfate on the left side. In the third surgery, the insertion site of materials was the same as the first surgery.

**Figure 1:**
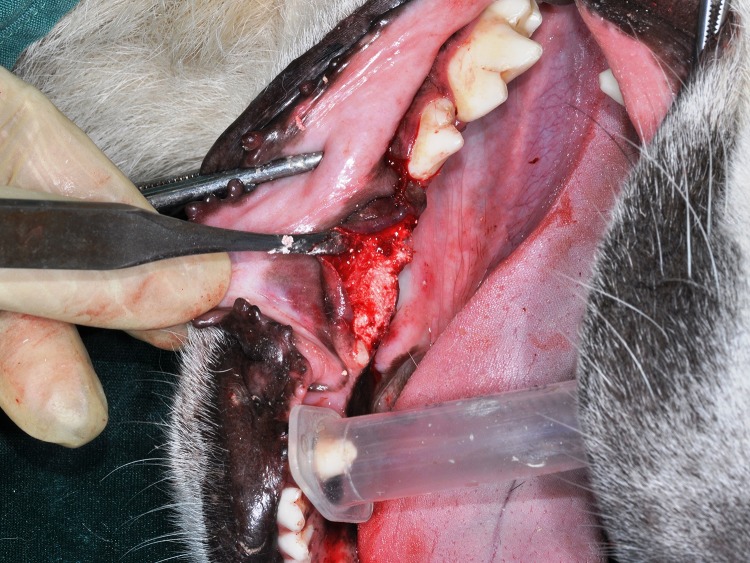
The sockets of the second and third mandibular premolars filled with hemihydrate calcium sulfate granules.

**Figure 2:**
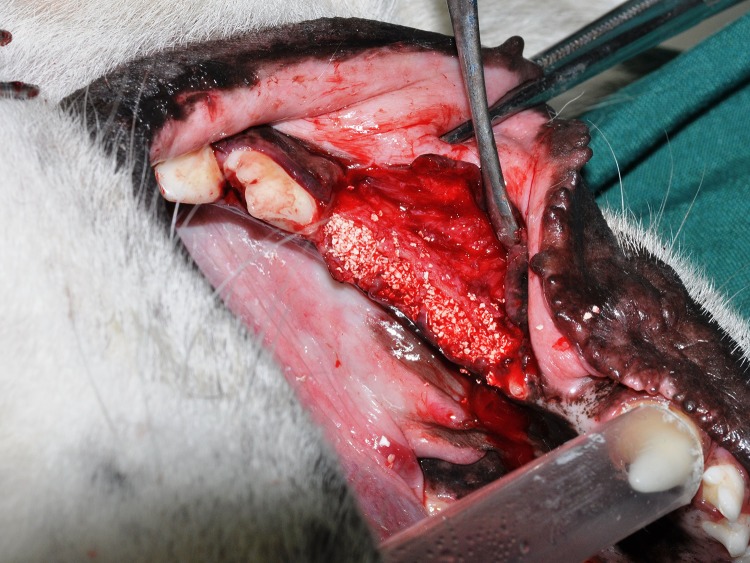
The sockets of the second and third mandibular premolars filled with Cerabone.

After inserting the materials into the cavities (Figures 1 and 2), the tissue flaps without using a membrane were repositioned using tension-free adaptation and interrupted absorbable sutures.

### Postoperative care

After the surgery, antibiotics (Cefazolin vial, 1 gr [produced by Loghman Co. of Iran], twice a day for three days, and Amikacin injection, 500 mg/2 mL [produced by Caspian Tamin Co. of Iran] at 20 mg/kg for three days) and analgesics (Meloxicam, 0.2 mg/kg, once a day for three days) were administered systemically. The animals received soft diet for one week to facilitate soft tissue repair.

### Sacrificing animals

After four and eight weeks, the dogs were sacrificed with respect to ethical principles with a 1-gr Thiopental sodium vial (produced by Exir Co. of Iran). Then, all the tissues of the tongue, lips, and cheeks were cut off by incisions below the mucogingival line. Finally, the jaw blocks were prepared from the mesial surface of the first premolar to the distal surface of the fourth premolar in each half of the lower jaw. For preparing histological sections, the aforementioned blocks were fixated in 10% buffered formalin after removing soft tissue coating.

### Sample processing steps

After transferring the samples to the histopathology laboratory, the blocks were placed in 10% nitric acid solution for four weeks for decalcification, and this solution was refreshed every day. After softening of the samples, washing (24 hours) in running water and storage (24 hours) in 5% sodium sulfate solution were performed to neutralize residual acid in the samples. To remove water, the samples were first placed in 70% ethanol for three hours, followed by three hours in 90% ethanol, three hours in 96% ethanol, and three times in pure ethanol, each for six hours.

**Figure 3:**
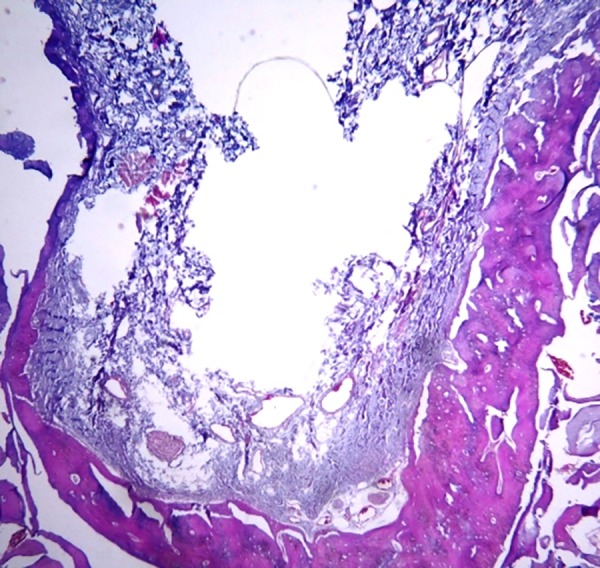
The buccolingual section of a socket 8 weeks after surgery.

The clearing process of the samples was performed by replacing ethanol with xylene, and then the samples were immersed in molten paraffin so that paraffin replaced xylene in the tissues. The samples were then placed in paraffin blocks to prepare them for sectioning. After these steps, from each paraffin block, eight sections (4 µm in thickness) were prepared using a microtome. Sections were then mounted on slides, and stained in hematoxylin and eosin (H&E) for histological examination (Figure 3). Unfortunately, in one of the samples in which the tooth cavities were filled with Cerabone, and the sample source dog was killed after four weeks, processing steps were not passed properly for unknown reasons and, as a result, we could not provide suitable slides for histological examination.

### Histological examination

Histological examination of the slides obtained from all the samples was performed by a pathologist using a Leica binocular microscope equipped with a Sony digital color camera. The samples were examined by a blinded pathologist. Histomorphometric measurements of the slides were carried out using ImageJ software developed at the National Institutes of Health. The measurements consisted of the ratio of the area of newly formed bone to the area of the entire cavity, and the ratio of the area of fibrous connective tissue to the area of the entire cavity. The slides were also examined for the presence or absence of inflammation.

### Statistical analysis

The hypotheses were tested using non-parametric Wilcoxon signed-rank test and McNemar test. Sign test was used in cases in which the required assumptions for Wilcoxon signed-rank test were not met. The statistical significance level was considered at P<0.05.

## Results

**Figure 4:**
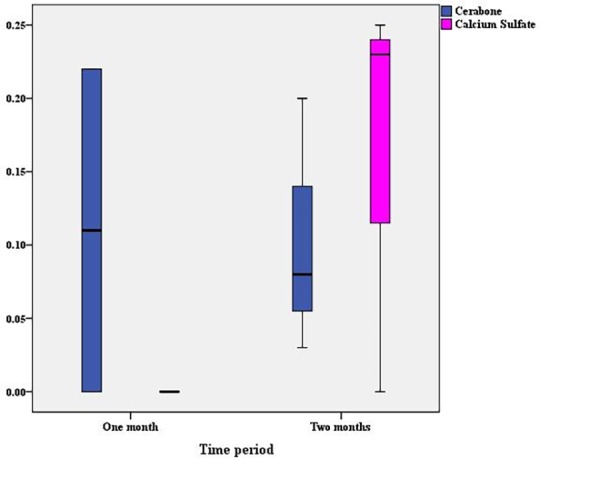
The distribution of the ratio of the area of newly formed bone to the area of the entire cavity in the Cerabone and calcium sulfate groups at 1-month and 2-month intervals

Comparison of the ratio of the area of newly formed bone to the area of the entire cavity showed that although the mean of the Cerabone group was slightly higher than that of the calcium sulfate group, no statistically significant difference was observed (Table 1). The separate comparison of the ratio of the area of newly formed bone to the area of the entire cavity in one-month (P=0.32) and two-month (P≈1.00) periods did not show any statistically significant differences between the two groups of Cerabone and calcium sulfate (Figure 4). In the two-month period, unlike in other cases, the average ratio of the area of newly formed bone to the area of the entire cavity in the calcium sulfate group was slightly higher.

**Table 1:** Comparison of the ratio of the area of newly formed bone to the area of the entire cavity between the Cerabone and calcium sulfate groups

	**No.**	**Mean**	**SD**	**Minimum**	**Maximum**	**Percentiles**			**Z** ^*^	**P-value**
						**25** ^th^	**50** ^th^ **(Median)**	**75** ^th^		
**Cerabone**	5	0.11	0.10	0.00	0.22	0.02	0.08	0.21	-0.55	0.58
**Calcium sulfate**	6	0.08	0.12	0.00	0.25	0.00	0.00	0.24		

* Wilcoxon Signed Ranks Test

**Figure 5:**
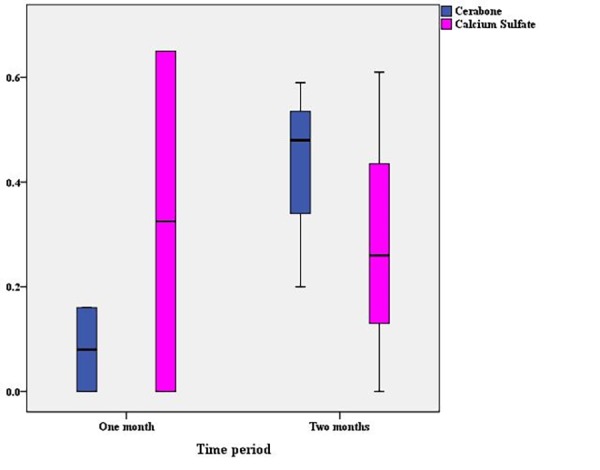
The distribution of the ratio of the area of fibrous connective tissue to the area of the entire cavity in the Cerabone and calcium sulfate groups at 1-month and 2-month intervals

Comparison of the ratio of the area of fibrous connective tissue to the area of the entire cavity showed that although the mean of the calcium sulfate group was slightly higher than that of the Cerabone group, no statistically significant difference was observed (Table 2). Separate comparison of the ratio of the area of fibrous connective tissue to the area of the entire cavity at 1-month (P=0.32) and 2-month (P≈1.00) intervals did not show any statistically significant differences between the Cerabone and calcium sulfate groups (Figure 5). At the 2-month interval, unlike in other cases, the average ratio of the area of fibrous connective tissue to the area of the entire cavity in the Cerabone group was slightly higher.

**Table 2:** Comparison of the ratio of the area of fibrous connective tissue to the area of the entire cavity between the Cerabone and calcium sulfate groups

	**No.**	**Mean**	**SD**	**Minimum**	**Maximum**	**Percentiles**			**Z** ^*^	**P-value**
						**25** ^th^	**50** ^th^ **(Median)**	**75** ^th^		
**Cerabone**	5	0.29	0.24	0.00	0.59	0.08	0.20	0.54	-0.37	0.72
**Calcium sulfate**	6	0.33	0.29	0.00	0.65	0.00	0.37	0.62		

* Wilcoxon Signed Ranks Test

Examination of the slides for the presence or absence of inflammation showed no inflammation in the Cerabone group samples, while 50% of the samples in the calcium sulfate group (three samples) were inflamed. However, this difference was not statistically significant (Table 3). In the calcium sulfate group, at 1-month interval approximately 67% of the samples (two samples), and at the 2-month interval approximately 33% of the samples (one sample) showed inflammation. The separate comparison of the two groups of Cerabone and calcium sulfate at 1-month and 2-month intervals also showed no statistically significant difference (Table 3).

**Table 3:** The distribution of samples in terms of the presence or absence of inflammation in the Cerabone and calcium sulfate groups overall and at 1-month and 2-month intervals

**Time period**	**Cerabone**	**Calcium sulfate**		**P-value** ^*^
		**Without inflammation**	**With inflammation**	
**All**	Without inflammation	3	2	0.50
	With inflammation	0	0	
**One month**	Without inflammation	1	1	1.00
	With inflammation	0	0	
**Two month**	Without inflammation	2	1	1.00
	With inflammation	0	0	

*McNemar Test

## Discussion

The formation of the alveolar process is dependent on teeth, and it is gradually resorbed following tooth loss. Bone loss due to tooth extraction might make it difficult to reconstruct or perform implantation.^20^ Socket preservation can play an important role in the maintenance of the alveolar process, and facilitates treatments such as implantation.^21^ For socket preservation, different methods have been suggested, involving the use of various bone grafts.^6^ The results of this study on the histological and histomorphometric comparison of the effects of calcium sulfate and Cerabone on socket preservation in dogs showed that these two substances at 4-week and 8-week intervals were not significantly different regarding bone formation, fibrous connective tissue and inflammation.

Orsini et al^22^ histologically evaluated the effects of calcium sulfate on the reconstruction of bone defects in the tibia of rabbits after two and four weeks. The results showed the presence of calcium sulfate after two weeks, and the newly formed bone had filled about 10% of the defects. After four weeks, calcium sulfate was almost completely resorbed and replaced with new bone. Approximately 34% of the defects were filled at this time by the newly formed bone. Additionally, no inflammatory cells were present in the samples. In another study by Ruga et al,^23^ histological evaluation of the intrasocket tissue in 19 sockets filled with calcium sulfate during tooth extraction showed that after three months 63.16% of the tissue was newly formed vital bone, 2.1% was non-vital bone, 4.74% was fibrous tissue and 30% was amorphous material. In addition, no calcium sulfate was left, and no inflammatory reaction was observed. Sbordone et al^24^ placed calcium sulfate in the sockets of maxillary canines. After five months, surgery was performed to place an implant in the same area. Evaluations showed that complete filling of the cavity with newly formed dense bone had occurred. Histological examination confirmed complete resorption of the bone graft and its replacement with newly formed bone.

In our study, the mean ratio of the area of newly formed bone to the area of the entire cavity was 8% for the calcium sulfate group and 11% for the Cerabone group. Since the bone density of the mandible of dogs is much higher than that of humans^25^ and we had to separate the roots during the teeth extraction, the periodontal ligament (PDL) was removed. In human dental sockets, PDL is wider and bone density is lower, and thus tooth extraction is associated with less surgical injury. On the other hand, in our study, several adjacent dental sockets were created, which could be effective in our results compared to the condition of a single-root tooth extraction. These issues affect early-stage bone formation and might lead to different results in our study compared to the above-mentioned studies.

In relation to Cerabone, in addition to the traumatic conditions mentioned above, it can be said that xenograft particles enclosed in connective tissue, and covered with multinucleated cells have been observed in the early stages (two weeks) of the reconstruction of bone defects containing a xenograft. Meanwhile, bone defects without any bone grafts, by then, represented newly formed woven bone which occupied most of the cavity.^26^ This response is a foreign body reaction that is observed with xenografts, although they do not clinically produce an immune response, are non-toxic and chemically neutral, and only lead to a delayed repair response during the earliest stages of the reconstruction.^27^ Furthermore, even after six months or more, 20−40% of the xenograft remains.^28^ In our study, due to the short duration of the study (8 weeks), there was not enough time to observe osteogenesis by Cerabone, and only in small areas of the cavities newly formed bone was observed.

Our examination of the presence or absence of inflammation showed no evidence of inflammation in any the Cerabone group cases, while 50% of the calcium sulfate group samples showed inflammation. Of course, this difference was not statistically significant. In some other studies using calcium sulfate for the repair of bone defects, there has been no evidence of inflammation, in contrast to this study.^22,23,29^ On the other hand, in a study by Sargolzaie et al^19^ on the preparation of hemihydrate calcium sulfate granules and its histological evaluation, the results were consistent with the present study and revealed inflammation. In that study, calcium sulfate granules were prepared from pure calcium sulfate powder and a binder. The results showed the presence of inflammatory cells in the third and sixth months, but there were no signs of inflammation in the 9th, 12th, 14th and 16th months. In their study, even in the presence of inflammation, no symptoms of sequestrum were observed, and osteogenesis was detectable from the third month. In our study, the inevitable injury during tooth extraction and the presence of the binder used in the preparation of calcium sulfate granules (the same granules prepared in the study of Sargolzaie et al) might have led to inflammatory reactions. However, this inflammatory reaction did not result in necrosis and bone sequestrum.

## Conclusion

The results of this study on histological and histomorphometric comparisons of the effects of calcium sulfate and Cerabone on socket preservation in dogs showed that these two materials were not significantly different at 4- and 8-week intervals regarding bone formation, fibrous connective tissue and inflammation. Considering the small number of samples, the above results should be interpreted with caution.

## Authors’ contributions

The study was planned by NS, MR and HK. NS, MR and HSS conducted the surgeries. Samples processing, and histological examination of the slides were performed by RZM. Statistical analyses and interpretation of data were carried out by HK. The manuscript was prepared by MR, and edited by NS, HSS, RZM and HK. All the authors have read and approved the final manuscript for submission.

## Acknowledgments

This article was originated from a postgraduate thesis registered under #606 at the Academic Affairs Office of School of Dentistry, Mashhad University of Medical Sciences.

## Funding

This research was supported by a grant from Mashhad University of Medical Sciences.

## Competing interests

The authors declare that they have no competing interests with regards to authorship and/or publication of this article.

## Ethics approval

All animal experiments in this study were performed in accordance with the Ethical Committee for Animal Care and Use of Mashhad University of Medical Sciences, Ethical Committee Acts (ethical approval code: IR.mums.sd.REC.1394.77). Also, all animal procedures were performed in accordance with NIH animal care guidelines.
